# Formaldehyde Emissions From 2‐Octyl Cyanoacrylate: Quantified Risk for Allergic Contact Dermatitis

**DOI:** 10.1111/cod.70110

**Published:** 2026-02-16

**Authors:** Daniel S. Rouhani, Paul M. Villalobos, Steven Zeng, Reza Ghodsi, Kavana M. Sanjay, Simrin A. Singh, M. Mark Mofid

**Affiliations:** ^1^ Department of Surgery, Division of Plastic and Reconstructive Surgery University of California San Francisco San Francisco California USA; ^2^ Department of Neurological Surgery University of California San Francisco San Francisco California USA; ^3^ Department of Plastic and Reconstructive Surgery Johns Hopkins University School of Medicine Baltimore Maryland USA; ^4^ Zilkha Neurogenetic Institute, Department of Physiology and Neuroscience, Keck School of Medicine University of Southern California Los Angeles California USA; ^5^ San Diego Skin, Inc. La Jolla California USA

**Keywords:** allergic contact dermatitis, cyanoacrylate, Dermabond Prineo, formaldehyde, skin adhesives

## Abstract

**Background:**

2‐octyl‐cyanoacrylate‐mesh‐systems are used as topical wound closure devices in surgery and are increasingly associated with allergic and irritant contact dermatitis. Formaldehyde, a degradation byproduct of cyanoacrylates and a potent sensitiser, is believed to be associated with this reaction, but emission levels have not been quantified.

**Methods:**

A bench microchamber study was performed using standardised 4 × 4 cm polyester mesh with applied 2‐octyl cyanoacrylate adhesive in a 30 L chamber (25°C, 1 atm, 1 h^−1^ air exchange). Formaldehyde off‐gassing was captured with DNPH cartridges (NIOSH 2016) and quantified via HPLC‐UV over 2 h. Data were upscaled to simulate clinical use and projected to 14 days under constant‐flux and flux‐halving scenarios.

**Results:**

Dermal loads at 14 days reached 8.4 μg/cm^2^, over eight times the EPA 1 μg/cm^2^ benchmark and above the 4.5–7.5 μg/cm^2^ elicitation range reported in formaldehyde‐sensitive patients. Under flux‐halving conditions, dermal exposure remained at 4.8 μg/cm^2^, nearly five times the EPA benchmark and above the elicitation threshold. Mean 2‐h airborne concentrations of 0.020–0.0385 ppm exceeded the NIOSH limit of 0.016 ppm by approximately 1.25–2.41‐fold.

**Conclusion:**

2‐octyl‐cyanoacrylate‐mesh‐systems emit formaldehyde at levels exceeding sensitization and inhalation thresholds, raising concern for dermal and respiratory exposure, particularly in sensitised individuals. Prior sensitisation from adhesives such as nail, eyelash or super glues may heighten risk.

## Graphical abstract

This study found that 2‐octyl cyanoacrylate skin adhesives release formaldehyde at levels up to 8.4× above EPA dermal thresholds and up to 2.41× above NIOSH airborne limits, producing exposures sufficient to provoke allergic and irritant contact dermatitis in patients.

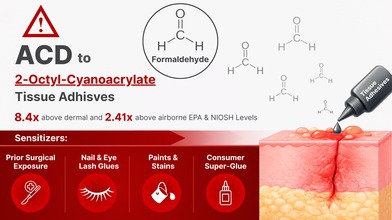

## 1 Introduction

### 1.1 History of Cyanoacrylates

Cyanoacrylate adhesives have undergone significant evolution since their initial development, transitioning from industrial glues to medical‐grade products widely used in surgical and emergency settings [1, 2]. In 1998, the US Food and Drug Administration (FDA) approved 2‐octyl cyanoacrylate (2‐OCA) (Dermabond) for topical skin closure [3]. However, clinical data from the initial 510(k) application showed higher suspected infection rates in Dermabond treated patients (3.6%) than in the two suture control groups (0.9% and 1.2%), representing roughly four‐fold and three‐fold increases, respectively. In 2012, Dermabond Prineo was introduced as a mesh‐reinforced adhesive system combining a polyester mesh impregnated with a benzalkonium chloride catalyst and a pen‐style applicator for the Dermabond 2‐OCA adhesive [4, 5]. Extensive clinical evidence has linked the use of these adhesives to serious adverse events [6, 7, 8], most notably allergic contact dermatitis (ACD), a type IV hypersensitivity reaction that can lead to vesiculobullous reactions, systemic erythema and an increased risk of surgical site infections (SSIs) [9].

### 1.2 Formulations of Cyanoacrylates

All cyanoacrylates share the general chemical formula of CH_2_=C(CN)CO_2_R, where the variable alkyl side chain R determines attributes such as strength, flexibility, curing times and toxicity (Figure S1) [10, 11]. Upon contact with anions, typically moisture, cyanoacrylates undergo rapid polymerisation through an exothermic reaction into polycyanoacrylates and form a rigid network [12]. Short‐chain ethyl cyanoacrylates used in industrial applications cure in under 10 s, yet yield brittle films and release large amounts of formaldehyde unsuitable for human skin [13]. A longer four‐carbon side chain, as in n‐butyl‐2 cyanoacrylate (n‐BCA), increases cure time to 30 s, decreasing the release of toxic byproducts. The most commonly used cyanoacrylate in medicine is 2‐octyl cyanoacrylate (2‐OCA). Its extended eight‐carbon side chain allows for slower polymerisation (60–90 s), improved flexibility and reduced formaldehyde release compared to earlier industrial formulations [14]. Commercial names and formulations for common cyanoacrylate adhesives are presented in Table 1.

**TABLE 1:** Common commercial and medical cyanoacrylate adhesives.

Cyanoacrylate formulation	Commercial product	Cure time^a^	Mechanical profile	Allergens & sensitivities	Indications for use
Ethyl cyanoacrylate	Loctite Super Glue^b^ Krazy Glue^c^ Gorilla Super Glue^d^	≤ 10 s	Glass‐hard, very brittle; high exotherm	High formaldehyde liberation, highly toxic; hydroquinone stabiliser; cross‐reactivity with nail glues, eyelash adhesives, acrylic paints	Not FDA‐cleared for skin closure (industrial/household adhesive)
n‐Butyl‐2 cyanoacrylate	LiquiBand Flow Control^e^ Histoacryl^f^	30 s	Rigid film	Low formaldehyde liberation; D&C Violet #2 dye; cross‐reactivity with nail glues, eyelash adhesives, acrylic paints	FDA‐cleared topical skin adhesive for low‐tension incisions/lacerations
2‐Octyl cyanoacrylate	Dermabond^g^ Exofin High‐Viscosity^h^ Derma+Flex QS^h^ LiquiBand Exceed^e^ LiquiBand Rapid^e^ SurgiSeal^i^	60–144 s	Flexible yet strong film	Low formaldehyde liberation; D&C Violet #2 dye; cross‐reactivity with nail glues, eyelash adhesives, acrylic paints	FDA‐cleared topical skin adhesive for clean, low‐tension incisions/lacerations
2‐Octyl cyanoacrylate + mesh system	Dermabond Prineo^g^ Exofin Fusion^h^ LiquiBand XL^e^	~60 s	Composite film with distributed tensile support	Low formaldehyde liberation; D&C Violet #2 dye; benzalkonium chloride; cross‐reactivity with nail glues, eyelash adhesives, acrylic paints	FDA‐cleared adhesive‐mesh system for surgical incisions ≤ 20 cm
Blend	Indermil Flexifuze^j^	~60 s	Highly elastic film	Low formaldehyde liberation; D&C Violet #2 dye; cross‐reactivity with nail glues, eyelash adhesives, acrylic paints	Not FDA‐cleared in the United States (CE‐mark only)

^a^ Cure times are based on standard lab or manufacturer testing (typically ~23°C for consumer glues, ~32°C–37°C for medical adhesives). Real‐world results may vary depending on temperature, humidity and substrate.

^b^ Henkel Corporation, Rocky Hill, CT, USA.

^c^ Elmer's Products Inc., Columbus, OH, USA.

^d^ The Gorilla Glue Company, Cincinnati, OH, USA.

^e^ Advanced Medical Solutions plc, Winsford, Cheshire, UK.

^f^ B. Braun Melsungen AG, Melsungen, Germany.

^g^ Ethicon Inc. (Johnson & Johnson), Raritan, NJ, USA.

^h^ Chemence Medical Inc., Alpharetta, GA, USA.

^i^ H.B. Fuller Medical Adhesive Technologies LLC, Maplewood, MN, USA.

^j^ Connexicon Medical Ltd., Dublin, Ireland.

### 1.3 Polymerisation and Chemical Reaction Pathways for Cyanoacrylates

**FIGURE 1:**
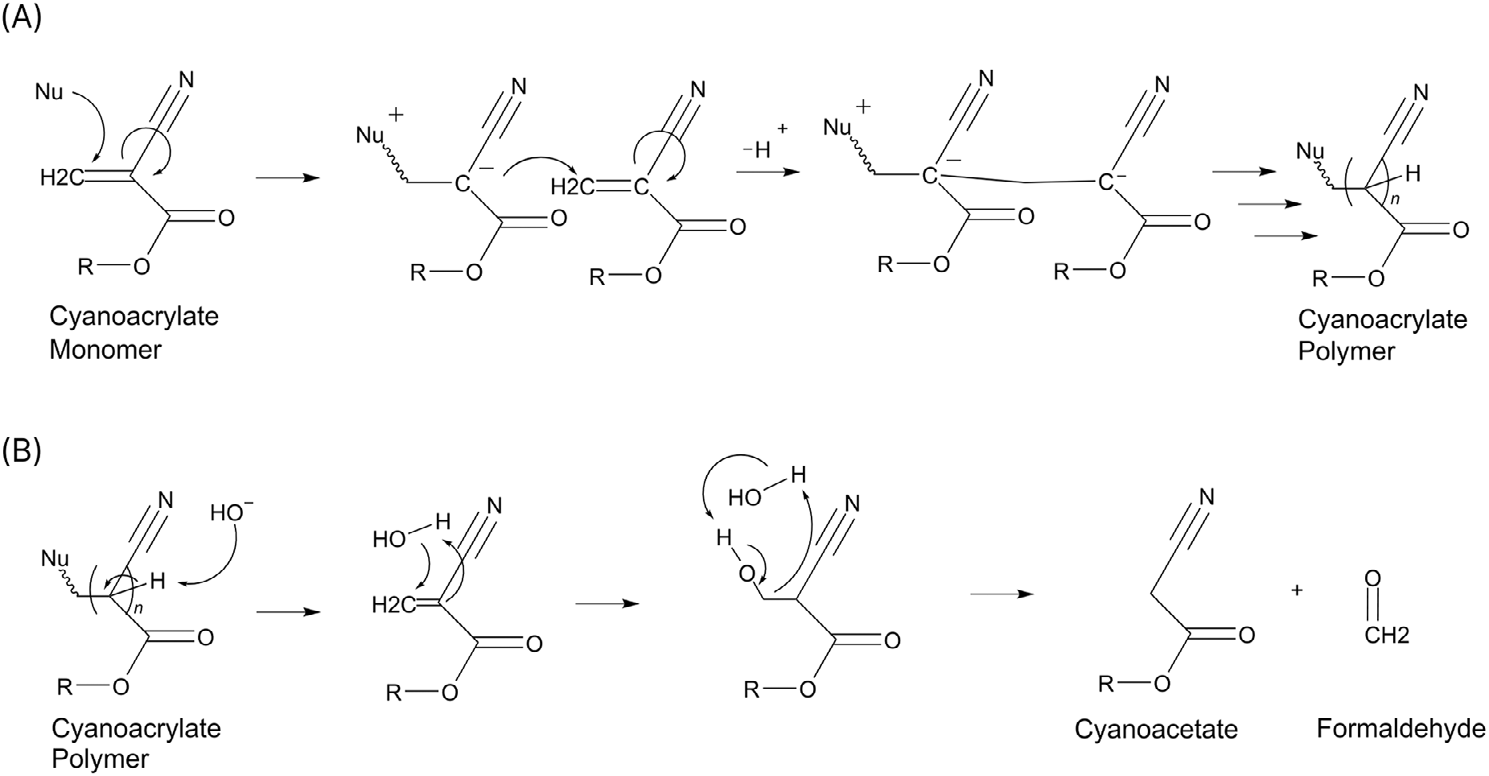
Polymerisation and degradation pathways of 2‐octyl cyanoacrylate. (A) depicts nucleophile [Nu] initiated polymerisation of cyanoacrylate monomers, where an anion, such as water, may attack the carbon–carbon double bond, creating a rapid, repetitive linkage of nearby cyanoacrylate monomers. (B) depicts hydrolytic cleavage of the polymer and a subsequent retro‐aldol pathway of degradation into cyanoacetate and formaldehyde.

The polymerisation and subsequent degradation of cyanoacrylates occur through distinct chemical pathways. Upon cyanoacrylate adhesive application, the most readily available anion, water, will neutralise the acid stabiliser, attack the monomer and trigger a cascade of exothermic reactions, where each monomer will subsequently attack the next, quickly forming a polymer network as illustrated in Figure 1A [9, 12].

During degradation, the polymer can undergo hydrolytic scission followed by retro‐Knoevenagel hydrolysis, as illustrated in Figure 1B, producing cyanoacetate and formaldehyde [15]. The nitrile and ester groups significantly withdraw electrons from the central carbon, creating an opening for hydroxyl ions to attack the carbon–carbon linkages, with possible sites at the end or in the middle of these polymer chains [16]. As an allergen and carcinogen, the formation of formaldehyde is a particular concern, appearing directly on the surface of the skin [9]. This behaviour supports the understanding that formaldehyde is generated through two main processes: (i) the breakdown of any unreacted monomers and (ii) the gradual degradation of the polymer itself.

Several constituents and degradation products of 2‐OCA mesh systems may contribute to allergic or irritant contact dermatitis, including the cyanoacrylate monomer, (meth)acrylates, dyes, benzalkonium chloride and formaldehyde [17]. Clinical patch‐test series in patients with ACD to 2‐OCA adhesives consistently identify cyanoacrylate monomers as the most frequent allergens, with formaldehyde‐positive tests occurring less often [17, 18, 19]. Although the role of cyanoacrylate monomers as primary sensitisers is well described, the contribution of formaldehyde as a co‐sensitiser or irritant remains uncertain. Formaldehyde is a well‐established contact allergen in the general patch‐tested population, has low elicitation thresholds, and is generated directly at the skin–adhesive interface as 2‐OCA polymerises and degrades [17, 20]. Device labeling for 2‐OCA adhesives specifically warns against its use in individuals with known hypersensitivity to cyanoacrylate, formaldehyde or benzalkonium chloride [18]. Quantifying formaldehyde emission from 2‐OCA mesh systems therefore addresses an important gap in the current literature.

### 1.4 Toxicity and Allergic Contact Dermatitis

**FIGURE 2:**
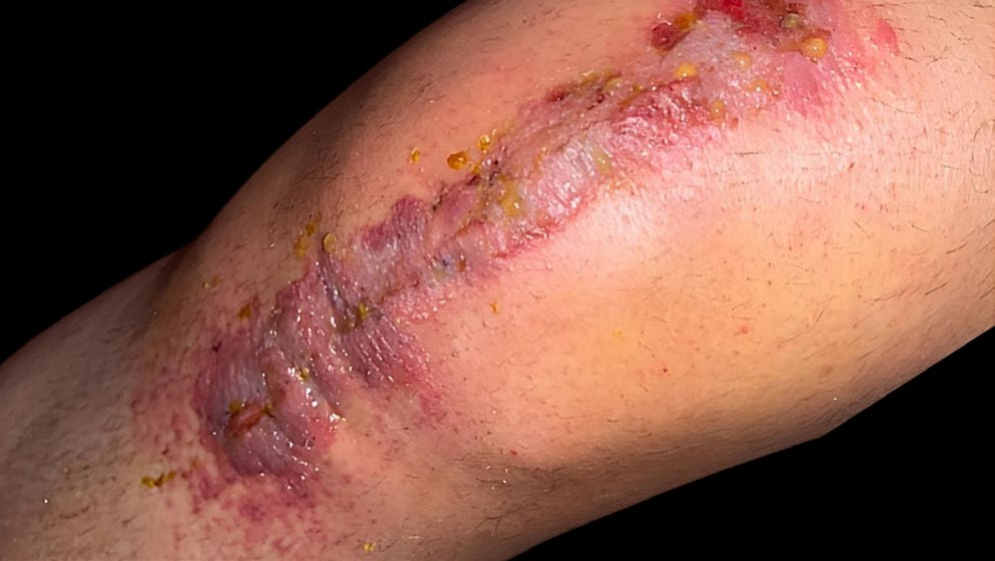
Severe allergic contact dermatitis to the dermabond prineo skin closure system. Following total knee arthroplasty, severe allergic contact dermatitis is evident along the incision after mesh removal, characterised by marked erythema, oedema and vesicle formation.

ACD is a delayed type IV hypersensitivity reaction, initiated by the activation of T‐cells and other immune mediators (Figure 2) [19]. Degradation products like cyanoacrylate monomers and formaldehyde can bind to skin proteins, forming haptens that are taken up by antigen‐presenting cells such as macrophages, ultimately triggering a localised inflammatory immune response [19]. Furthermore, formaldehyde is classified as an IARC Group 1 carcinogen, demonstrating deleterious effects on cell health, such as genotoxicity and apoptosis, through any means of exposure [21].

Human elicitation thresholds for formaldehyde induced ACD are low. In occluded patch‐test studies of formaldehyde‐sensitised patients, Flyvholm et al. [22] reported a no‐observed‐adverse‐effect level (NOAEL) of approximately 1.5 μg/cm^2^ and a lowest‐observed‐adverse‐effect level (LOAEL) of 7.5 μg/cm^2^, while Fischer et al. [23] reported a LOAEL of 4.5 μg/cm^2^. These values together define an elicitation range of approximately 4.5–7.5 μg/cm^2^ for sensitised individuals. Although formaldehyde is volatile on open skin, 2‐octyl cyanoacrylate adhesives cure into a solid film construct, within minutes of application, and can limit outward loss and concentrate exposure at the skin interface. For this reason, occluded patch‐test thresholds are a clinically relevant benchmark for comparison. Regulatory assessments by the United States Environmental Protection Agency (EPA) have incorporated this human data, together with animal evidence and safety factors, to derive a conservative health‐protective skin‐loading benchmark of 1 μg/cm^2^ [24]. We therefore use these reference ranges to evaluate modelled dermal exposures in this study. In addition to dermal exposure, the National Institute for Occupational Safety and Health (NIOSH) recommends a limit of 0.016 ppm for airborne formaldehyde, which serves as our inhalation benchmark [25].

This study provides the first quantitative assessment of formaldehyde release from 2‐OCA mesh systems, conducted under testing conditions aligned with the US National Institute for Occupational Safety and Health (NIOSH) standards. We hypothesise that the degradation of 2‐OCA mesh systems generate measurable dermal and airborne formaldehyde exposures that exceed current health‐protective limits and reach levels capable of triggering ACD or irritant contact dermatitis and potential sensitisation in individuals.

## 2 Materials and Methods

### 2.1 Test Materials

Microchamber testing was commissioned by the Center for Environmental Health (Oakland, CA) and performed independently by Applied Technical Services LLC (Marietta, GA) using ASTM D7706 and NIOSH Method 2016. Two replicate 4 × 4 cm 2‐OCA mesh samples were prepared and evaluated under identical conditions to allow duplicate emission measurements. The DRM001 Dermabond Prineo skin closure system, comprising a polyester mesh impregnated with benzalkonium chloride and a separate applicator containing 2‐OCA adhesive, was maintained in its original sterile packaging until testing. To accommodate the microchamber dimensions, 4 × 4 cm mesh samples were excised from the larger full samples. A thin layer of 2‐OCA adhesive, consistent with standard clinical practice, was applied to each mesh section. All components were weighed prior to assembly. Polymerisation and formaldehyde release were initiated by adding deionised water to the aluminium dish in the setting‐controlled microchamber.

### 2.2 Microchamber Arrangement

Formaldehyde emissions were quantified in a 30 L microchamber operated under controlled conditions (25°C, 1 atm, 1 h^−1^ air exchange) for the duration of testing. Emission sampling followed ASTM D7706 guidelines, outlining a screening technique for volatile organic compounds on indoor materials [26]. Clean air was introduced at a constant flow to permit off‐gassing. Formaldehyde was captured using 2,4‐dinitrophenylhydrazine (DNPH)‐coated silica gel sampler cartridges per NIOSH Method 2016 and measured through high‐performance liquid chromatography (HPLC)‐UV [27]. Controlled airflow brings formaldehyde into contact with DNPH, which reacts and produces a stable derivative that can then be quantified via chromatography. Emission samples were collected over 2 h.

### 2.3 Upscaling and Exposure Modelling

Attributes of the 16 cm^2^ samples were scaled proportionally to the surface area of full‐size 2‐OCA mesh systems used in clinical practice. Each of the sizes, 22 × 4 cm (88 cm^2^), 60 × 2 cm (120 cm^2^) and 42 × 4 cm (168 cm^2^) represents a 5.5, 7.5 and 10.5‐fold increase, respectively, which was used to estimate corresponding mesh and 2‐OCA masses. All calculations were repeated on both samples and then averaged between the two.

Surface‐specific formaldehyde concentrations were estimated based on the ratios of mass emission over known surface areas to calculate dermal load. Surface density (*D*) was computed as $D=\frac{m}{A}$, where *m* is formaldehyde mass in μg and *A* is area in cm^2^ [22]. The model assumes uniform release across the adhesive surface, full skin contact and accumulation of formaldehyde over the time period. Because 2‐octyl cyanoacrylate rapidly polymerises into a solid film, the interface may behave as an occluded microenvironment with limited outward volatilisation [28]. To conservatively account for potential outward loss of formaldehyde (e.g., diffusion from edges or the outer surface during curing), we also report a retained‐fraction sensitivity case assuming 50% of generated formaldehyde contributes to skin‐interface loading.

We extended the 2‐h chamber measurements to 48 h and then to 7, 10 and 14 days, based on the emission kinetics reported by Pascual et al. [16] for cyanoacrylate adhesives, where cumulative formaldehyde release was tracked from 0 through 38 days and showed a sustained, approximately linear increase during the early period, with n‐butyl cyanoacrylate still rising linearly at day 38 [16]. To examine a more conservative case where emissions slow after the initial curing period, we modelled a scenario in which the 2‐h flux stayed constant through 48 h, then was reduced by 50% for the remainder of the wear time. Airborne formaldehyde concentrations measured in the 30 L, 1 h^−1^ microchamber were normalised to adhesive surface area. We did not extrapolate these concentrations linearly over time as in a ventilated chamber, concentrations approach a steady‐state determined by emission rate, chamber volume and ventilation. Final concentrations (μg/m^3^) were converted to parts per million (ppm) using the ideal gas law and formaldehyde‐specific constants at standard temperature and pressure (25°C, 1 atm): a molar volume of 24.45 L/mol and a molar mass of 30.03 g/mol [29].

## 3 Results

### 3.1 Formaldehyde Emissions From Adhesive Mesh Systems

In two replicate 4 × 4 cm (16 cm^2^) 2‐OCA mesh samples, total sample masses were 0.30–0.31 g and microchamber testing yielded formaldehyde concentrations of 4.3 and 4.7 μg/m^3^ and mass‐normalised emissions of 2.5 and 2.9 μg/g of sample. Thus, both samples released a mean of 2.7 μg formaldehyde per gram of material, corresponding to approximately 0.8 μg total formaldehyde per 16 cm^2^ sample and a mean 2‐h surface density of 0.05 μg/cm^2^ (Table S1).

### 3.2 Upscaled Dermal Loading of Formaldehyde to 2‐Octyl‐Cyanoacrylate & Polyester Mesh

**FIGURE 3:**
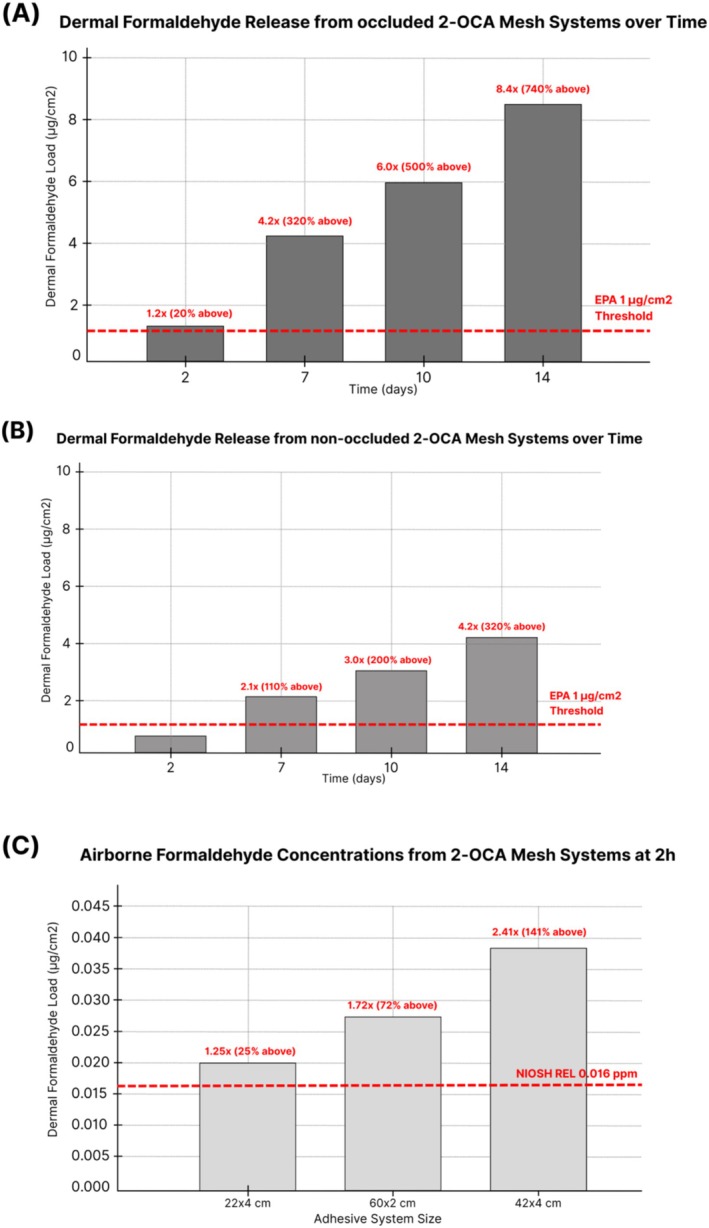
Dermal and airborne formaldehyde exposure from 2‐OCA mesh systems relative to health‐based benchmark. (A) Dermal surface formaldehyde loading on skin over time for a 42 × 4 cm occluded 2‐octyl‐cyanoacrylate mesh system under a constant‐flux model. Bars show modelled dermal doses at 2, 7, 10 and 14 days (1.2×, 4.2×, 6.0× and 8.4× the EPA health‐protective benchmark of 1 μg/cm^2^, corresponding to 20%, 320%, 500% and 740% above the benchmark). By 10–14 days, modelled loads enter and exceed the 4.5–7.5 μg/cm^2^ elicitation range reported for formaldehyde‐sensitised patients. (B) Dermal surface formaldehyde loading on skin for a 42 × 4 cm non‐occluded 2‐octyl‐cyanoacrylate mesh system under a constant‐emission flux model, in which 50% of generated formaldehyde is assumed to be lost to outward diffusion/volatilisation before reaching the skin–adhesive interface. Dermal doses surpassed the EPA health‐protective benchmark of 1 μg/cm^2^ at 2, 7 and 14 days (2.1×, 3.0× and 4.2×, corresponding to 110%, 200% and 320% above the benchmark, respectively). (C) Airborne formaldehyde concentrations measured in a 30 L microchamber 2 h after application of full‐size 2‐OCA mesh systems (22 × 4 cm, 60 × 2 cm and 42 × 4 cm). Bars show mean concentrations of 0.020, 0.0275 and 0.0385 ppm, respectively, with the red dashed line indicating the NIOSH Recommended Exposure Limit (REL) of 0.016 ppm. These correspond to approximately 1.25×, 1.72× and 2.41× the REL (about 25%, 72% and 141% above the limit).

These standardised results were then scaled to full 2‐OCA mesh system sizes (22 × 4 cm, 60 × 2 cm, 42 × 4 cm), which maintained similar adhesive mass per unit area and therefore similar 2‐h surface densities (0.05 μg/cm^2^ across configurations, Table S2). Because dermal load was expressed per unit area, modelled doses differed only slightly between sizes; we therefore report values for the 42 × 4 cm system as a representative upper bound. Under the constant‐flux model, in which the 2‐h flux is maintained throughout wear, cumulative dermal doses in this configuration reached 1.16–1.31 μg/cm^2^ at 48 h across the two samples, already above the 1 μg/cm^2^ EPA health‐protective benchmark. By 7, 10 and 14 days, modelled doses based on the mean 2‐h surface density increased to approximately 4.2, 6.0 and 8.4 μg/cm^2^, respectively, leading to exposures above the 4.5–7.5 μg/cm^2^ elicitation range (Figure 3A and Table S3). In the more conservative model, where the initial flux is maintained through 48 h and then reduced by 50%, cumulative surface densities in the 42 × 4 cm systems were 2.7 μg/cm^2^ at 7 days, 3.6 μg/cm^2^ at 10 days and 4.8 μg/cm^2^ at 14 days, approximately 3–5 times higher than the EPA health‐protective level and, by 14 days, still above the lowest elicitation dose of 4.5 μg/cm^2^ observed in human patch‐test studies (Table S3). Under the 50% retained‐fraction sensitivity case to account for non‐occluded potential volatilisation, 14‐day skin‐interface loads were 4.2 μg/cm^2^ (constant‐flux) and 2.4 μg/cm^2^ (flux‐halving) (Table S4). These values remain 2.4–4.2× above the EPA 1 μg/cm^2^ benchmark (Figure 3B).

### 3.3 Upscaled Airborne Loading of Formaldehyde to 2‐Octyl‐Cyanoacrylate & Polyester Mesh

Mean modelled airborne formaldehyde concentrations exceeded the NIOSH Recommended Exposure Limit (REL) of 0.016 ppm by approximately 1.25–2.41‐fold, with mean predicted levels of 0.020, 0.0275 and 0.0385 ppm for the 22 × 4 cm, 60 × 2 cm and 42 × 4 cm configurations, respectively (Figure 3B). Across duplicate runs, 2‐h concentrations ranged from 0.019–0.021 ppm for the 22 × 4 cm configuration, 0.026–0.029 ppm for the 60 × 2 cm configuration and 0.037–0.040 ppm for the 42 × 4 cm configuration (Table S2) [30].

## 4 Discussion

### 4.1 Dermal and Gaseous Formaldehyde Emissions

In this study, modelled dermal formaldehyde doses from all 2‐OCA mesh adhesive systems substantially exceeded levels that are generally considered acceptable for sensitised skin. Under a constant‐flux assumption, 14‐day surface densities reached approximately 8.4 μg/cm^2^, about eight times higher than the 1 μg/cm^2^ health‐protective skin loading used in regulatory risk assessments by the EPA and above the 4.5–7.5 μg/cm^2^ human elicitation range [22, 23]. Even in a more conservative scenario, modelled 14‐day doses reached 4.8 μg/cm^2^, nearly five times higher than the EPA health‐protective benchmark and just above the lowest human elicitation dose of 4.5 μg/cm^2^.

These findings indicate that, over a typical 7–14 day wear period, 2‐OCA mesh systems can deliver a sustained formaldehyde burden at the wound surface that is not only well above EPA's health‐protective level, but also approaches or enters the human elicitation band in patients with formaldehyde allergy. Because formaldehyde is a powerful trigger of type IV hypersensitivity reactions, even small increases in dermal exposure can significantly raise the risk of allergic contact dermatitis [31]. In routine clinical practice, clinicians may apply 2‐OCA mesh systems to the same or different areas of the body, potentially leading to cumulative formaldehyde exposure and an increased risk of local or systemic allergic reactions [32]. In addition to sustained accumulation at the adhesive–skin interface, formaldehyde is also released into the surrounding environment from the adhesive–air interface, creating a secondary vector of exposure via inhalation. In our 30 L microchamber, mean 2‐h airborne concentrations in the upscaled systems were 0.020–0.0385 ppm, corresponding to approximately 1.25–2.41 times the NIOSH recommended exposure limit of 0.016 ppm. Peak values up to 0.040 ppm were observed in the largest system and overlap with concentrations (0.019 ppm) reported to cause symptoms in hospital workers [30]. This vapour can migrate into the patient's or provider's breathing zone [33], creating a continuous inhalational co‐exposure during early wear or during the application of the adhesive [34, 35].

Repeated open application tests (ROATs) may provide a useful point of comparison as they represent the effect of volatilisation of formaldehyde [36]. In a double‐blind ROAT study, Hauksson et al. [37] applied approximately 0.1 g of a 2000‐ppm formaldehyde moisturiser twice daily for a maximum of 4 weeks to a 5 × 5 cm area (a load of 8 μg/cm^2^ per application), and nine of 17 formaldehyde‐sensitive participants developed eczematous reactions, whereas none occurred in controls. Under our most conservative assumption (flux‐halving after 48 h with only 50% retained at the skin interface), the modelled 14‐day adhesive formaldehyde load of 2.4–4.2 μg/cm^2^ corresponds to roughly one‐half of a single ROAT application‐equivalent. This indicates that a clinically meaningful formaldehyde burden can persist at the skin–adhesive interface even when substantial outward loss is assumed.

### 4.2 Safety and Prevention

Repeated exposure to 2‐OCA has been strongly associated with an increased incidence of ACD, consistent with a delayed‐type (Type IV) hypersensitivity mechanism. In a prospective cohort of 234 paediatric orthopaedic procedures, Koritz et al. reported an initial ACD rate of 12% following a single Dermabond application. Among children who underwent subsequent procedures involving re‐exposure to 2‐OCA, the incidence rose to 21%, reflecting a 75% relative increase [38]. In a prospective study of hip and knee arthroplasty patients, those without prior exposure to a polyester mesh and 2‐OCA adhesive developed ACD at a rate of 1.9%, compared to 8.1% in those with prior exposure (*p* < 0.01), which represents a 4.3‐fold increase [39]. The effect was most pronounced in staged bilateral total knee arthroplasty, where the incidence of ACD rose from 1% after the first unexposed procedure to 22% after the second re‐exposed knee (*p* < 0.001), representing a 22‐fold increase [40].

This same sensitisation pathway extends beyond clinical re‐exposures. Cyanoacrylates are widely used in consumer products, including household super glues, model cements, fast‐drying paint primers and cosmetic adhesives for artificial nails and eyelashes [41]. Community contact with these products can similarly prime memory T‐cell responses [42]. A case by Sato et al. describes a 24‐year‐old woman who developed ACD after Dermabond use, then experienced a similar reaction months later to both a commercial eyelash adhesive and a household glue [43].

Humidity is another key factor which mediates the curing of 2‐OCA, as water acts as the nucleophile that initiates anionic polymerisation. In low‐humidity environments, polymerisation is slowed, leading to prolonged skin contact with the unreacted monomer and increasing the production of degradation byproducts such as formaldehyde. Bitterman et al. documented a case of ACD following Dermabond application, with the reaction possibly exacerbated by the use of a space heater that reduced ambient humidity [44]. Similarly, Bowen et al. reported several cases of ACD associated with 2‐OCA localised in the more arid states in the United States [45].

2‐OCA mesh systems such as Dermabond Prineo amplify this reaction via benzalkonium chloride, a quaternary ammonium compound embedded in the polyester mesh as an accelerator. While faster curing shortens monomer skin contact, it also intensifies the exothermic reaction and may increase thermal and chemical degradation of the polymer, which is also capable of releasing formaldehyde. Most notably, benzalkonium chloride itself is a known irritant [45]. In a 2667‐patient patch‐test series at Mayo Clinic (2017–2021), benzalkonium chloride ranked among the 15 allergens with the highest reaction rates, underscoring its growing relevance as a contact allergen [46, 47].

### 4.3 Limitations and Future Directions

A key limitation of this study is that formaldehyde emissions were measured using a standardised microchamber protocol under controlled laboratory conditions, rather than on human skin or in dynamic clinical environments. While this method provides consistent airflow, temperature, and follows national guidelines, it does not fully capture the variability introduced by anatomical contours, wound exudate, adhesive pooling or partial occlusion. Nonetheless, the 2‐hour DNPH sampling window was purposefully selected based on established NIOSH and ASTM guidelines to capture the early post‐curing emission rate, before occlusion and dressing interactions dominate. This design allowed us to isolate and separately model formaldehyde flux from the skin–adhesive interface (representing trapped dermal exposure under mesh occlusion) and the adhesive–air interface (representing airborne exposure during application and early wear). Future studies should investigate how variations in application technique, adhesive thickness and environmental conditions influence total formaldehyde exposure, both at the skin interface and over time.

## 5 Conclusion

This study provides the first quantitative assessment of formaldehyde emissions from the Dermabond Prineo skin closure system. In our testing, estimated dermal loading of formaldehyde over a typical 14 day wear period reached approximately 8.4 μg/cm^2^, about eight‐fold higher than the 1 μg/cm^2^ EPA health‐protective benchmark and above the 4.5–7.5 μg/cm^2^ range that has elicited reactions in formaldehyde‐sensitised patients. Mean 2‐h airborne concentrations of 0.020–0.0385 ppm exceeded the NIOSH limit of 0.016 ppm by approximately 1.25–2.41‐fold. Formaldehyde vapour released during curing can present an additional hazard for both the patient and the provider during application and drying, and when sealed under dressings can create a toxic microenvironment that compounds the overall systemic burden. Given that sensitisation to cyanoacrylates is often acquired through prior exposure to both medical adhesives and widely used consumer products, such as nail glues, eyelash adhesives, super glues and paints, subsequent surgical application can provoke severe allergic contact dermatitis. Repeated or cross‐reactive exposures may also contribute to broader population‐level sensitisation which is a major public health concern. Together, these growing yet preventable risks highlight the necessity of re‐evaluating the place of cyanoacrylate skin adhesives in contemporary surgical practice.

## Author Contributions

**Daniel S. Rouhani:** investigation, writing – original draft, writing – review and editing, visualization, validation, methodology, formal analysis, project administration, supervision. **Paul M. Villalobos:** writing – original draft, writing – review and editing, visualization. **Steven Zeng:** writing – original draft, visualization, supervision, writing – review and editing. **Reza Ghodsi:** writing – original draft, writing – review and editing, formal analysis, methodology. **Kavana M. Sanjay:** writing – review and editing, writing – original draft, project administration, methodology. **Simrin A. Singh:** writing – original draft, writing – review and editing, visualization. **M. Mark Mofid:** writing – original draft, writing – review and editing, investigation, conceptualization, methodology, validation, visualization, formal analysis, project administration, data curation, supervision, resources.

## Funding

Product testing was commissioned by and paid for by the Center for Environmental Health (Oakland, CA).

## Consent

Written patient consent for publication of identifiable clinical images was obtained and is on file.

## Conflicts of Interest

Daniel S. Rouhani serves as a consultant to Sylke, Inc. M. Mark Mofid, MD is an officer and a shareholder of Sylke, Inc.

## Supporting information


**Figure S1:** Cyanoacrylate molecular composition and corresponding family based on alkyl groups.
**Table S1:** Dermabond prineo testing information and formaldehyde emission quantities, over 2‐h testing period.
**Table S2:** Upscaled formaldehyde dermal loading and airborne emission rates to standard Dermabond prineo sizes.
**Table S3:** Modelled cumulative dermal formaldehyde surface loading (μg/cm^2^) for a 42 × 4 cm Dermabond Prineo mesh system under two emission scenarios.
**Table S4:** Sensitivity analysis of modelled skin‐interface formaldehyde loading (42 × 4 cm system).

## Data Availability

The data that supports the findings of this study are available in the Supporting Information of this article.
