# Supplementary material for: Formaldehyde Emissions From 2‐Octyl Cyanoacrylate: Quantified Risk for Allergic Contact Dermatitis
Source: Contact Dermatitis. 2026 Feb 16;94(6):635–44. doi: 10.1111/cod.70110 (PMC13139693; doi:10.1111/cod.70110)
Supplement: Supplementary file 1 — Figure S1: Cyanoacrylate molecular composition and corresponding family based on alkyl groups. Table S1: Dermabond prineo testing information and formaldehyde emission quantities, over 2‐h testing period. Table S2: Upscaled formaldehyde dermal loading and airborne emission rates to standard Dermabond prineo sizes. Table S3: Modelled cumulative dermal formaldehyde surface loading (μg/cm2) for a 42 × 4 cm Dermabond Prineo mesh system under two emission scenarios. Table S4: Sensitivity analysis of modelled skin‐interface formaldehyde loading (42 × 4 cm system). [file COD-94-635-s001.docx]

**eFigure1. Cyanoacrylate Molecular Composition and Corresponding Family Based on Alkyl Groups**


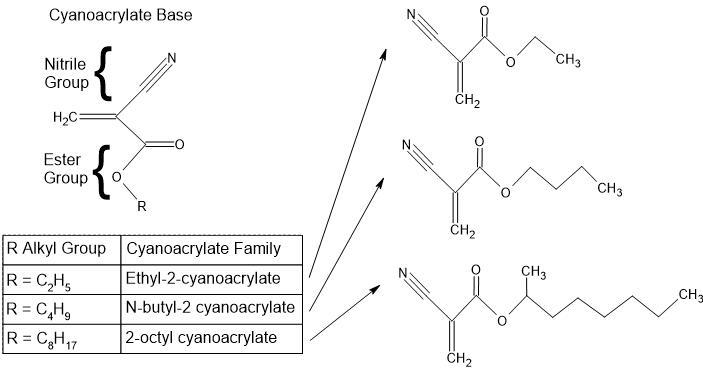


Ethyl-2 cyanoacrylate [C_6_H_7_NO_2_], N-butyl 2 cyanoacrylate [C_8_H_11_NO_2_], and 2-octyl cyanoacrylate [C_12_H_19_NO_2_]. Adapted from Isaac & Thiemer, 1975.

***e*Table 1**. **Dermabond Prineo Testing Information and Formaldehyde Emission Quantities, over 2-hour Testing Period.**

| **Sample** | **Mesh Mass (g)** | **Mesh Area (cm)** | **CA^a^ Mass (g)** | **FA^b^ Air Concentration (μg/m^3^)** | **FA per Sample (μg/g)** | **FA Surface Density (μg/cm^2^)** | **Total FA Release (μg)** |
| --- | --- | --- | --- | --- | --- | --- | --- |
| 1 | 0.05 | 4 x 4 | 0.26 | 4.3 | 2.5 | 0.05 | 0.78 |
| 2 | 0.05 | 4 x 4 | 0.25 | 4.7 | 2.9 | 0.05 | 0.87 |

1. *CA = 2-octyl cyanoacrylate*
2. *FA = formaldehyde.*

**eTable 2. Upscaled Formaldehyde Dermal Loading and Airborne Emission Rates to Standard Dermabond Prineo sizes.**

| **Source Sample** | **Dermabond Prineo Size** | **CA^a^ mass (g)** | **Total FA^b^ Release (μg) 2h** | **FA Surface Density (μg/cm^2^) 2h** | **FA Air Quantity (μg/m^3^) 2h** | **FA PPM 2h** |
| --- | --- | --- | --- | --- | --- | --- |
| 1 | 22 x 4 cm | 1.43 | 4.26 | 0.05 | 23.65 | 1.93E-02 |
|  | 60 x 2 cm | 1.95 | 5.81 | 0.05 | 32.25 | 2.63E-02 |
|  | 42 x 4 cm | 2.73 | 8.14 | 0.05 | 45.15 | 3.68E-02 |
| 2 | 22 x 4 cm | 1.38 | 4.79 | 0.05 | 25.85 | 2.10E-02 |
|  | 60 x 2 cm | 1.88 | 6.53 | 0.05 | 35.25 | 2.87E-02 |
|  | 42 x 4 cm | 2.63 | 9.14 | 0.05 | 49.35 | 4.02E-02 |

a) CA = 2-octyl cyanoacrylate

b) FA = Formaldehyde

**eTable 3. Modeled cumulative dermal formaldehyde surface loading (µg/cm^2^) for a 42 x 4 cm Dermabond Prineo mesh system under two emission scenarios.**

| \| **Time since application** \| \| --- \|  \|  \| \| --- \| | \| **Constant-flux modelᵃ (µg/cm²)** \| \| --- \|  \|  \| \| --- \| | \| **Conservative modelᵇ (µg/cm²)** \| \| --- \|  \|  \| \| --- \| |
| --- | --- | --- | --- | --- | --- | --- | --- | --- |
| 2 days | 1.2 | 1.2 |
| 7 days | 4.2 | 2.7 |
| 10 days | 6.0 | 3.6 |
| 14 days | 8.4 | 4.8 |

ᵃ Constant-flux model assumes the 2-hour surface density of 0.05 µg/cm² corresponds to a constant emission flux maintained throughout the wear period.
ᵇ Conservative model assumes the 2-hour flux is maintained through 48 hours, then decreases by 50% for the remainder of wear.

**eTable 4. Sensitivity analysis of modeled skin-interface formaldehyde loading (42 × 4 cm system)**

| **Model assumption** | **48 hours** | **7 days** | **10 days** | **14 days** |
| --- | --- | --- | --- | --- |
| **Constant emission fluxᵃ; full retention at skinᶜ** | **1.16-1.31** | **4.2** | **6.0** | **8.4** |
| **Flux halves after 48 hᵇ; full retention at skinᶜ** | **1.16-1.31** | **2.7** | **3.6** | **4.8** |
| **Constant emission fluxᵃ; 50% retained at skinᵈ** | **0.58-0.66** | **2.1** | **3.0** | **4.2** |
| **Flux halves after 48 hᵇ; 50% retained at skinᵈ** | **0.58-0.66** | **1.35** | **1.8** | **2.4** |

ᵃ Constant emission flux refers to maintaining the 2-hour measured formaldehyde release rate throughout the wear period.
ᵇ “Flux halves after 48 h” represents a conservative scenario in which formaldehyde release slows after the initial curing period.
ᶜ “Full retention at skin” assumes that all formaldehyde generated at the adhesive-air interface contributes to skin-interface loading.
ᵈ “50% retained at skin” assumes that half of the generated formaldehyde is lost to outward diffusion/volatilization before reaching the skin-adhesive interface.
